# Rosanortriterpenes A–B, Two Promising Agents from *Rosa laevigata* var. *leiocapus*, Alleviate Inflammatory Responses and Liver Fibrosis in In Vitro Cell Models

**DOI:** 10.1155/2020/8872945

**Published:** 2020-11-10

**Authors:** Bai-Lin Li, Juan-Juan Hu, Jin-Dan Xie, Chen Ni, Hui-Jun Liang, Qian-Ran Li, Jie Yuan, Jie-Wei Wu

**Affiliations:** Guangdong Provincial Key Laboratory of New Drug Development and Research of Chinese Medicine, Mathematical Engineering Academy of Chinese Medicine, Guangzhou University of Chinese Medicine, Guangzhou 510006, China

## Abstract

Rosanortriterpenes A–B (RTA and RTB), two nortriterpenoids, are characteristic constituents in the fruits of *Rosa laevigata* var. *leiocapus*. However, pharmacological studies on these compounds are still scarce. In the present study, we aim to investigate the anti-inflammatory mechanisms associated with the effects of RTA–B in RAW264.7 macrophages and LO_2_ cells by detecting cell viabilities, nitric oxide (NO) production, tumour necrosis factor-alpha (TNF-*α*), and interleukin-6 (IL-6) production. Simultaneously, the anti-inflammatory action mechanisms of these two compounds were illustrated through western blot assay. Besides, the antihepatic fibrosis activities of these compounds have also been explored. The results demonstrated that RTA and RTB inhibited the production of NO, TNF-*α*, and IL-6 and suppressed liver fibrosis. RTA and RTB treatment also greatly inhibited the activation of NF-kappaB (NF-*κ*B) pathway. Our study confirmed the promising anti-inflammatory and anti-liver fibrosis actions of RTA–B, suggesting that they might be developed as alternative and promising drugs for the treatment of hepatic inflammatory and fibrotic diseases.

## 1. Introduction

Inflammation is an immune response caused by tissue damages or pathogen infections [1]. Excessive and uncontrolled inflammations are harmful to the body, especially liver, the largest metabolic organ in the human body, as they may cause chronic diseases and further lead to fatty liver diseases, hepatitis, liver fibrosis, and even cirrhosis and liver cancer [2–6]. Currently, the commercial drugs utilized to treat inflammation are mainly steroidal anti-inflammatory drugs (SAIDs) and nonsteroidal anti-inflammatory drugs (NSAIDs). However, with the increasing number of medical side effects discovered in clinical applications such as low potassium and hypertension [7–11], novel inflammatory drugs are urgently needed [12]. Traditional diets, in particular, edible plants, with pharmacological functions such as pigeon pea, orange peel, and grapefruit [13–15] have been always regarded as an essential source for treatment of inflammation. Therefore, it may be a potential way to seek new anti-inflammatory agents from traditional medicinal food [16].

*Rosa laevigata* belonging to the Rosaceae family is widely distributed throughout southern China. Its fruits are recorded in Chinese Pharmacopoeia as a treatment for spermatorrhea, uroclepsia, frequent micturition, and uterine abscission and hence considered as a kind of traditional medicinal food in East Asia [17]. *Rosa laevigata* var. *leiocapus*, a variety of *R. laevigata*, was firstly found and reported in Boluo county, Guangdong province, China, by Wang et al. in 1995 [18]. The specific morphological characteristics of this species attributed to its smooth fruits without any thorns. As a result of this distinction, *Rosa laevigata* var. *leiocapus* can be developed as an alternative to *R. laevigata*, which can improve production efficiency and save labor costs, since there is no need to remove thorns from the fruit during harvesting and processing.

**Figure 1:**
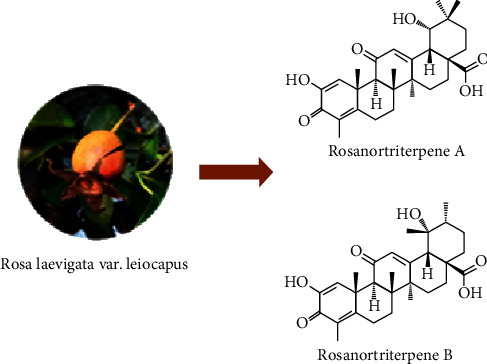
Structures of compounds RTA and RTB.

However, as characteristic constituents isolated from *Rosa laevigata* var. *leiocapus* fruits [19], the bioactivity evaluations of rosanortriterpenes A–B (RTA and RTB) (Figure 1) have rarely been reported. Therefore, studies on anti-inflammatory and antiliver fibrosis activities of RTA and RTB are presented in this paper.

## 2. Materials and Methods

### 2.1. Reagents

RTA and RTB were isolated from *R. laevigata* var. *leiocapus* and stored in several small aliquots at −80°C. Materials used for cell culture such as FBS (made in Australia), DMEM (made in China), PBS (pH 7.4), and penicillin/streptomycin were obtained from Gibco Company. The LPS (*Escherichia coli* 0127:B8), MTT powder, indomethacin (Indo), silibinin (SLB), and DMSO solvent were obtained from Sigma-Aldrich (St. Louis, MO, USA). TNF-*α* and TGF-*β* were obtained from PeproTech (Rocky Hill, NJ, USA). Griess reagent, DAF-FM DA, RIPA buffer, and BCA protein assay kits were purchased from Biotine (Shanghai, China). Nuclear protein extraction kits and Hoechst 33342 were purchased from Solarbio (Beijing, China). PVDF membranes were obtained from Millipore (Bedford, MA, USA). The NF-*κ*B pathway sampler kit and *β*-actin rabbit antibodies were brought from Cell Signaling Technology Company (Danvers, MA, USA). The collagen I antibody, alpha-SMA antibody, and the enhanced chemiluminescence kit were obtained from Affinity Biosciences (Cincinnati, OH, USA). TNF-*α* and IL-6 enzyme immunoassay kits were purchased from Dakewe Biotech (Shenzhen, China).

### 2.2. Cell Line Culture

The RAW264.7 and HSC-T6 cells were gained from the CAS Cell Bank in Kunming. The LO_2_ cells were purchased from the Chinese Academy of Sciences. The abovementioned cells were, respectively, cultured at 37°C in the DMEM medium system containing 10% FBS as well as 1% penicillin/streptomycin.

### 2.3. MTT Assays

Cytotoxicity assays were carried out utilizing a typical MTT method based on the standard protocol. To be brief, RAW264.7, LO_2_, and HSC-T6 cells were cultured in 96-well cell culture microplates with a density of 10^4^ cells per well for LO_2_ and HSC-T6 cells and 5 × 10^4^ cells per well for RAW264.7. After cell adhesion, 100 *μ*L of culture medium with established concentrations of RTA, RTB, or equal volume of DMSO replaced the original medium, and then, the cells were further incubated for 24 h. 20 *μ*L of MTT (5 mg/mL) was added to all wells and coincubated for 3 h. After removing the medium, DMSO (150 *μ*L) was subjected to the wells to dissolve the formazan crystals. The solution was monitored using a microplate reader (Thermo, Massachusetts, USA) at 490 nm.

### 2.4. NO Production Assay

RAW264.7 macrophages were dispensed in 96-well plates with a density of 5 × 10^4^ cells per well. After cell adhesion, different concentrations of RTA and RTB were added to the cell culture medium 2 hours before LPS (100 ng/mL) stimulation. After 24 h, 100 *μ*L of Griess reagents A and B were added to the removed cell culture medium, respectively. The absorbance of the mixture was detected at 540 nm using the aforementioned microplate reader.

### 2.5. Intracellular NO Assays

The fluorescence data of intracellular NO were also detected using a NO-sensitive fluorescence probe DAF-FM DA. Briefly, RAW264.7 cells were dispensed in 96-well microplates with a density of 5 × 10^4^ cells per well, and RTA and RTB were added to the cell culture medium 2 hours before LPS stimulation after adhesion. After 24 h incubation, cells were loaded with 10 *μ*M of DAF-FM DA for 40 min at 37°C, then cells were carefully rinsed with PBS three times, and the data were recorded with 495 nm as excitation wavelength and 515 nm as emission wavelength using a microplate reader (Thermo, Massachusetts, USA).

The fluorescence microscopy imaging of intracellular NO was recorded utilizing DAF-FM DA. Similarly, RAW264.7 murine macrophages were dispensed in 96-well microplates with a density of 5 × 10^4^ cells per well. After cell adhesion, RTA and RTB were added to the cell culture medium 2 hours before LPS stimulation. After 24 h incubation, cells were loaded with 10 *μ*M of DAF-FM DA for 40 min at 37°C [20]. Then, cells were carefully rinsed by PBS for three times and then incubated for 30 min with Hoechst 33342. Fluorescence was detected with a fluorescence microscope (BX-53, Olympus, Japan).

### 2.6. Western Blot Analysis

RAW264.7, LO_2_, and HSC-T6 cells were seeded in 6-well plates, and then different concentrations of RTA–B were added to cells 2 h before LPS (100 ng/mL), TNF-*α* (20 ng/mL), or TGF-*β* (10 ng/mL) stimulation. After treatment for 24 h, cells were collected to extract protein for further western blot analysis.

### 2.7. ELISA

The experimental protocol is based on the previous report; briefly, RAW 264.7 cells and LO_2_ were dispensed in 96-well plates, and RTA and RTB at prescribed concentrations were added to the culture medium 2 h prior to LPS or TNF-*α* stimulation [14]. After 24 h, productions of TNF-*α* or IL-6 in the cell culture medium were detected according to the instruction of manufacturers after dilution.

### 2.8. Statistical Analysis

The data in this work were expressed as mean ± standard deviation (SD). One-way analysis of variance (ANOVA) together with Tukey multiple comparison tests were performed with GraphPad Prism 5 software.

## 3. Results and Discussion

### 3.1. Anti-Inflammatory Activities of RTA and RTB

#### 3.1.1. Cell Viability

**Figure 2:**
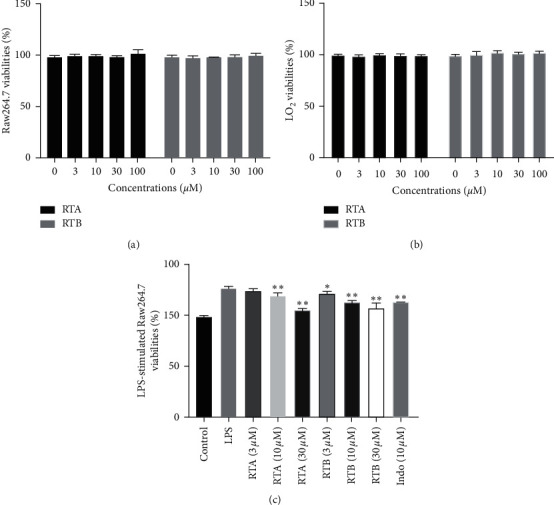
Effects of RTA and RTB on cell viabilities of (a) RAW264.7 and (b) LO_2_. ^*∗*^*p* < 0.05 and ^*∗∗*^*p* < 0.01 compound treatment groups vs. DMSO group. (c) Effects of RTA and RTB on cell viabilities of RAW264.7 stimulated by LPS (100 ng/mL). ^*∗*^*p* < 0.05 and ^*∗∗*^*p* < 0.01 compound treatment groups vs. LPS group.

Recruited myelo-derived free macrophages are important factors accounting for acute and chronic hepatitis [21]. Hence, two cell lines, RAW264.7 and LO_2_, were selected for the study on RTA and RTB for inflammation treatment. As a preliminary step, we tested their cytotoxicity against the two cells in 24 h treatment duration. As a result, even at the concentration of 100 *μ*M, viabilities of the two cells treated with both RTA–B were basically unchanged compared to the blank control group (Figures 2(a) and 2(b)), suggesting cytosafety of these compounds. In addition, we investigated the cell viability of LPS-stimulated RAW264.7 treated with RTA and RTB at the concentration of 3, 10, and 30 *μ*M with 10 *μ*M Indo acting as a positive control. The results are shown in Figure 2(c). 100 ng/mL LPS could stimulate the proliferation of RAW264.7; however, 24 h treatment by RTA and RTB could reduce the proliferation in a dose-dependent manner.

#### 3.1.2. NO Production Assay

**Figure 3:**
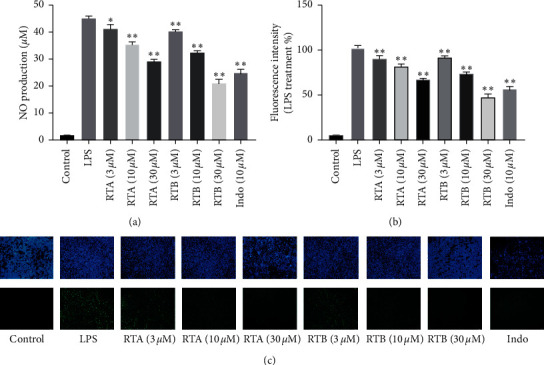
Effects of RTA–B on NO production. (a) Inhibitory effects of RTA and RTB on NO production of RAW264.7 murine macrophages stimulated with LPS (100 ng/mL). (b) Fluorescence data of intracellular NO of RAW264.7. ^*∗*^*p* < 0.05 and ^*∗∗*^*p* < 0.01 compound treatment groups vs. LPS group. (c) Intracellular NO microscopy imaging of RAW264.7, blue for the nucleus of living cells and green for intracellular NO with × 20 magnification.

RAW264.7 once stimulated by LPS will generate a great amount of NO which is one of the active radical species [22]. Excessive NO in return may greatly facilitate cascade reactions of inflammation [6, 23]. It has been widely accepted that LPS stimulation can significantly increase NO release in macrophages. In order to study the anti-inflammatory activities of RTA–B, we applied them to RAW264.7 cells stimulated by LPS to measure their effects on NO production and Indo was still chosen as a positive control.  Figure 3(a) displays a dosage-dependent NO production inhibition effects of the compounds. In terms of intracellular NO, consistent with the previous results, these compounds could inhibit the production of intracellular NO represented by the fluorescence intensity (Figure 3(b)). And as for intracellular NO microscopy imaging, for all groups, the blue fluorescence representing living cells stained by Hoechst 33342 remained almost stable. The control group showed weak green fluorescence representing intracellular NO. In contrast, cells treated by LPS exhibited strong green fluorescence. However, green fluorescence significantly weakened by RTA and RTB treatment implying their potential for anti-inflammatory activities (Figure 3(c)).

#### 3.1.3. Inhibition of NF-kB Activation by RTA and RTB

**Figure 4:**
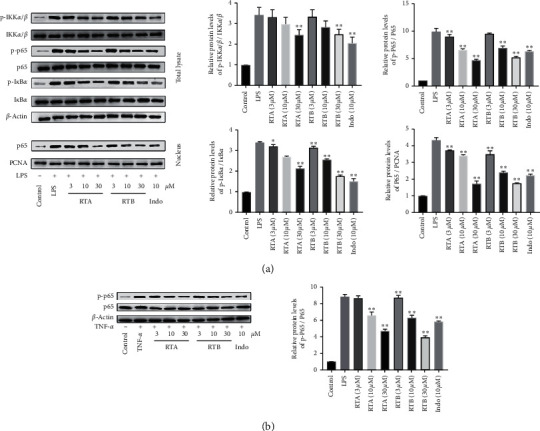
Detraction influences of RTA–B on protein expressions in NF-*κ*B pathway in (a) RAW264.7 cells stimulated by LPS (100 ng/mL) and (b) LO2 cells stimulated by TNF-*α* (20 ng/mL). ^*∗*^*p* < 0.05 and ^*∗∗*^*p* < 0.01 compound treatment groups vs. model group.

NF-*κ*B is one of the most significant signal pathways regulating proinflammatory genes whose productions in return give rise in the persistent activation of NF-*κ*B pathway and further exacerbate inflammatory response [24]. In resting cells, NF-*κ*B is normally present in the cytoplasm in formation as an inactive I*κ*B*α*/*p*50/*p*65 heterotrimer. Stimulated by activators such as LPS or TNF-*α*, IKK*α*/*β* is phosphorylated and activated IKK*α*/*β* triggers phosphorylation of I*κ*B*α* and release of p65 from the complex [25]. Dissociative p65 once translocated into the nucleus would initiate transcription of downstream genes inducing those coding proinflammatory cytokines [26]. To determine whether RTA and RTB achieve their anti-inflammatory effects via NF-*κ*B pathway, western blot analysis was launched to identify expressions of related proteins.  Figure 4(a) shows that compared with the blank control group, the productions of p-IKK*α*/*β* and p-p65 along with p-I*κ*B*α* in total lysate and p65 in the nucleus were significantly increased in the LPS-model group, while RTA and RTB remarkably suppressed the levels of these proteins in RAW264.7 cells. Similarly, the increase of p-p65 induced by TNF-*α* was also inhibited by RTA and RTB in LO_2_ cells. Consistent with our expectations, both RTA and RTB showed suppression of activated NF-*κ*B pathway both in macrophages and hepatic cells.

#### 3.1.4. Inhibition of TNF-*α* and IL-6 Production by RTA and RTB

**Figure 5:**
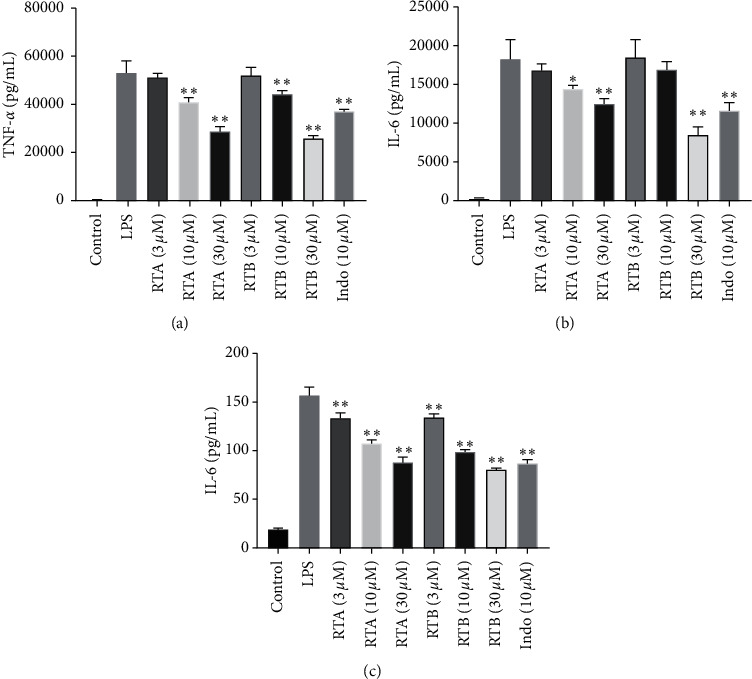
Detraction influences of RTA–B on (a) TNF-*α* in RAW264.7 stimulated by LPS (100 ng/mL), (b) IL-6 levels in RAW264.7 stimulated by LPS (100 ng/mL), and (c) IL-6 levels in LO_2_ stimulated by TNF-*α* (20 ng/mL). ^*∗*^*p* < 0.05 and ^*∗∗*^*p* < 0.01 compound treatment groups vs. model group.

TNF-*α* and IL-6 are two essential proinflammatory cytokines in inflammation, and their high expressions are commonly found in liver injuries [27–29]. In order to further clarify the anti-inflammatory effects of RTA and RTB, we detected the productions of these two proinflammatory cytokines in LPS-stimulated RAW264.7 and IL-6 in TNF-*α*-stimulated LO_2_. The results are displayed in Figure 5. The productions of TNF-*α* and IL-6 in the medium supernatant after treatment with different concentrations of RTA or RTB were lower than those only treated with LPS in a dose-dependent manner proving that these two compounds could reduce the productions of critical proinflammatory cytokines.

### 3.2. Antifibrotic Activities of RTA and RTB

#### 3.2.1. Cell Viability

**Figure 6:**
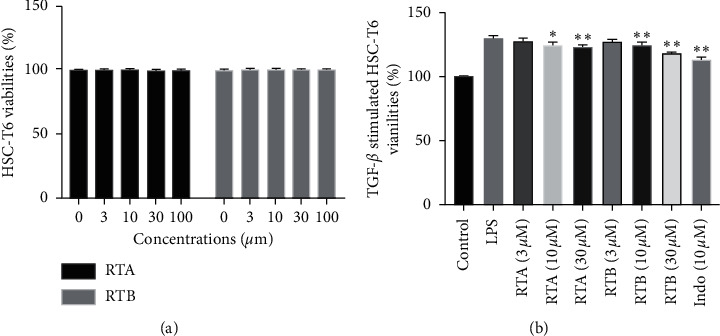
Effects of RTA and RTB on cell viabilities of (a) HSC-T6. ^*∗*^*p* < 0.05 and ^*∗∗*^*p* < 0.01 compound treatment groups vs. DMSO group. (b) Effects of RTA and RTB on cell viabilities of HSC-T6 stimulated by TGF-*β* (10 ng/mL). ^*∗*^*p* < 0.05 and ^*∗∗*^*p* < 0.01 compound treatment groups vs. TGF-*β* group.

In order to explore whether the compounds have antifibrotic activities in hepatic cells, we first tested their cytotoxicity against hepatic stellate cells, HSC-T6. Consistent with previous results, after 24 h treatment, viabilities of HSC-T6 treated by both compounds showed no significant differences compared to the blank control group even at the concentration of 100 *μ*M (Figure 6(a)). Additionally, we further investigated the cell viability of TGF-*β*-stimulated HSC-T6 treated by RTA and RTB at the concentration of 3, 10, and 30 *μ*M with 10 *μ*M SLB acting as a positive control. The results are shown in Figure 6(b). Stimulated by TGF-*β*, proliferation of HSC-T6 increased significantly; however, 24 h treatment by RTA and RTB could reduce the proliferation in a dose-dependent manner.

#### 3.2.2. Western Blot Analysis

**Figure 7:**
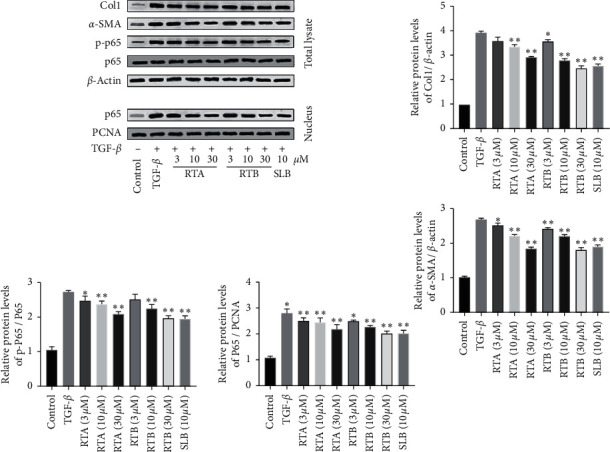
Influences of RTA–B on fibrosis-related protein expressions in HSC-T6 cells stimulated by TGF-*β* (10 ng/mL). ^*∗*^*p* < 0.05 and ^*∗∗*^*p* < 0.01 compound treatment groups vs. model group.

Liver fibrosis is characterized by excess deposition of extracellular matrix (ECM) such as collagen type-I (Col1) and *α*-smooth muscle actin (*α*-SMA) [30]. So we detected the effect of the compounds on the expression of the above proteins, and results are shown in Figure 7. After 24 h treatment, both RTA and RTB could inhibit the increase of ECM-related proteins stimulated by TGF-*β* in a dose-dependent manner. Besides, NF-*κ*B is also an important signaling pathway in the development of liver fibrosis that not only participates in the proliferation and apoptosis of liver cells but also promotes activation of hepatic stellate cells [31]. In HSC-T6 cells stimulated by TGF-*β*, the p-p65 was significantly increased, as well as p65 in nucleus. While under the treatment of the compounds, the levels of these two proteins decreased indicating that RTA and RTB could suppress NF-kB activation and ECM deposition induced by TGF-*β* in hepatic stellate cells.

## 4. Conclusions

In various cultures around the world, inflammation and relative diseases have always been treated by plant and plant-derived agents [32]. Among them, fruits of *Rosaceae* were reported to possess potential anti-inflammatory activities [33–35]. As a potential substitute for *R. laevigata*, the convenience of *Rosa laevigata* var. *leiocapus* in the food processing makes it more favorable to serve as a medicinal food source. In addition, the COVID-19 epidemic has seriously affected the world's political and economic patterns leading to food insecurity [36, 37]; hence, seeking and making full use of food resources with pharmacological values seems more significant in the current crisis. Thus, the medicinal values of *Rosa laevigata* var. *leiocapus* need to be determined urgently. In this present study, the anti-inflammatory and anti‐liver fibrosis effects of two characteristic compounds from *Rosa laevigata* var. *leiocapus*, RTA and RTB, were elaborated. Taking together, our work not only lays a foundation for the development and application of *Rosa laevigata* var. *leiocapus* but also provides dietary supplements targeting at liver inflammation and fibrosis.

## Data Availability

The materials and data presented in the work are available from the authors upon reasonable request.
